# Encoding Method of Single-cell Spatial Transcriptomics Sequencing

**DOI:** 10.7150/ijbs.43887

**Published:** 2020-07-30

**Authors:** Ying Zhou, Erteng Jia, Min Pan, Xiangwei Zhao, Qinyu Ge

**Affiliations:** 1 State Key Laboratory of Bioelectronics, School of Biological Science & Medical Engineering, Southeast University, Nanjing 210096, China.; 2 School of Medicine, Southeast University, Nanjing 210097, China.

**Keywords:** Single-cell RNA sequencing, Spatial transcriptomics, Encoding method, *In situ* sequencing

## Abstract

Despite significant advances in parallel single-cell RNA sequencing revealing astonishing cellular heterogeneity in many tissue types, the spatial information in the tissue context remains missing. Spatial transcriptome sequencing technology is designed to distinguish the gene expression of individual cells in their original location. The technology is important for the identification of tissue function, tracking developmental processes, and pathological and molecular detection. Encoding the position information is the key to spatial transcriptomics because different methods have different encoding efficiencies and application scenarios. In this review, we focus on the latest technologies of single-cell spatial transcriptomics, including technologies based on microwell plates, barcoded bead arrays, microdissection, *in situ* hybridization, and barcode *in situ* targeting, as well as mixed separation-based technologies. Moreover, we compare these encoding methods for use as a reference when choosing the appropriate technology.

## Introduction

About a century ago, Rudolph Virchow, who was the founder of modern pathology, postulated that all diseases originate in cells and thus started the cellular era 1. Tissues consist of multiple cell types and each type has a particular lineage and function that contributes to the organ's biology. What's more, subgroups of cells of the same type are usually genetically heterogeneous with each other as well as other cell types 2. As a result, single-cell sequencing has emerged to study the heterogeneity of cells. Differential expression of genes in various cell types with a common genome is a feature of complex cellular functions and the basis of multicellular organisms. Identifying the spatial organization of tissue at the cellular resolution from a single cell's gene expression profile is of great importance for understanding biological systems. In recent years, a number of single-cell RNA sequencing (scRNA-seq) methods have been developed, which have dramatically advanced in scale and power 3. Among them, methods based on plates are low throughput and distribute a single cell per well of multi-well plates. In contrast, methods based on beads are high throughput and sort a cellular suspension into a tiny droplet or a well that contains the reagent and a barcoded bead. What's more, combinatorial indexing methods are scalable without physically isolating individual cells 4.

**Figure 1:**
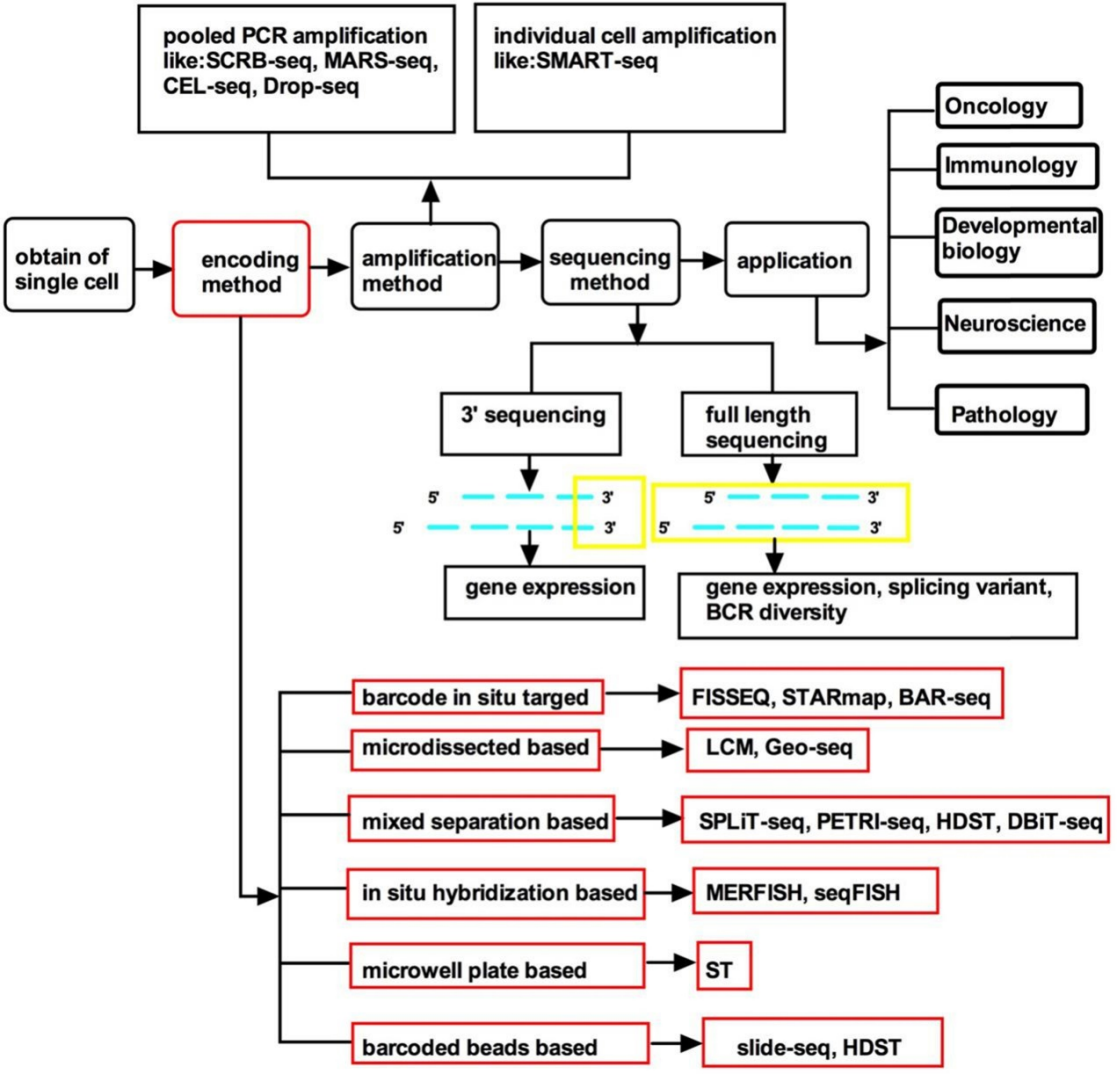
** Flow diagram of single-cell spatial transcriptomics.** The content discussed in this article is highlighted with a red border. Abbreviations: ST: spatial transcriptomics. HDST: high-definition spatial transcriptomics. SPLiT-seq: split-pool ligation-based transcriptome sequencing. Geo-seq: geographical position sequencing. UEI: unique event identifier. DBiT-seq: Deterministic Barcoding in Tissue for spatial omics sequencing. MERFISH: Multiplexed error-robust FISH. seqFISH: Sequential barcoded Fluorescence *in situ* Hybridization. FISSEQ: fluorescent *in situ* RNA sequencing. STARmap: spatially-resolved transcript amplicon readout mapping. BAR-seq: barcoded anatomy resolved by sequencing.

The spatial resolution of gene expression in complex biological tissues can be conventionally analyzed by *in situ* hybridization or immunohistochemistry 5. However, these technologies can only be used to analyze a small number of genes at one time with low throughput. Recently, advances in RNA sequencing have enabled the high-throughput analysis of the expression of many genes. The high throughput of RNA sequencing and the advantages of traditional spatially resolved technologies can be combined in spatial transcriptomics, which can collect mRNA data with a massive throughput. Spatial transcriptomics spatially and quantitatively detect differences in gene mRNA expression between separate tissue regions and enable a new type of bioinformatics analysis 6, 7. In addition, the gene expression of specific cells defines the state and type of the cells and the spatial organization which is closely related to the normal tissues' development and function, and thus also the pathogenesis and prognosis 8, 9. Therefore, the ability to perform single-cell profiling with spatially resolved transcriptomes will provide tissue biology with critical insights. There have already been some excellent reviews on single-cell spatial transcriptomics which have introduced the various technologies that can realize spatial transcriptomics 8, 10, the spatial transcriptomics of tissue-level systems 11, the challenges of spatial transcriptomics 12, the basic sample preparation and data analysis process 13, and how to use scRNA-seq and single-molecule fluorescence *in situ* hybridization (smFISH) to understand the brain 14. However, there is no article summarizing the specific field of the encoding methods of spatial transcriptomics. Single-cell spatial transcriptomic technologies include cell acquisition, location information encoding, amplification, cell library sequencing, and, finally, the application of the technology in different fields. The method of position information encoding is the key to spatial transcriptomics because different methods have different encoding efficiencies and applications. Here, we systematically summarize the encoding methods of state-of-the-art single-cell spatial transcriptomics and provide a comprehensive guide to choosing appropriate technologies (**Figure 1, Table 1**).

## Single-Cell Spatial Transcriptomics of Complete Samples

### Microwell plate-based technologies

**Figure 2:**
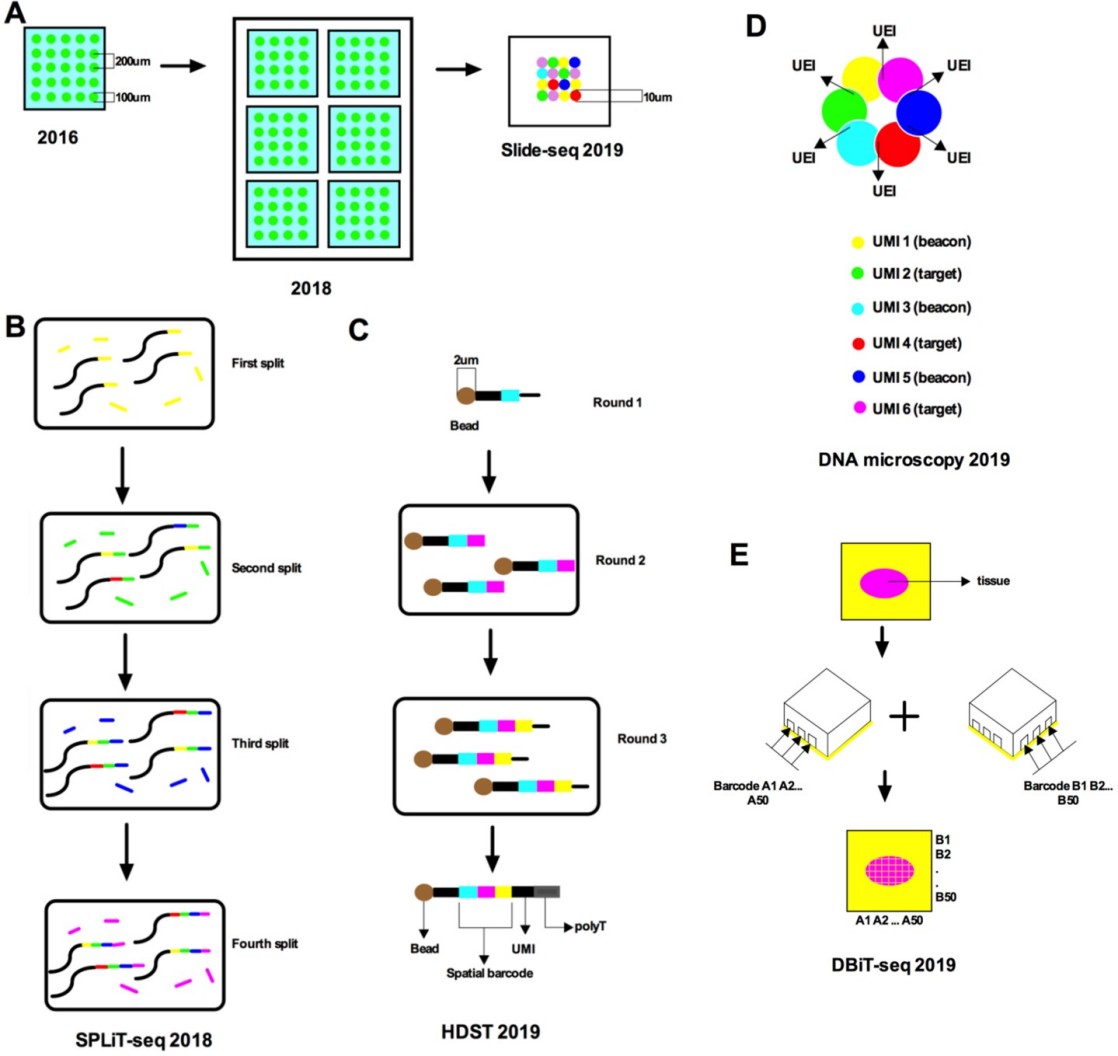
** Schematic of recent advances in single-cell spatial transcriptomics. A.** Stahl et al. (2016) used a barcoded microplate with a diameter of 100 μm and a center-to-center distance of 200 μm, over an area of 6.2 mm by 6.6 mm. Stahl et al. (2018) used barcoded oligo-dT microarray slides divided into six subarrays, each with a size of 6.2 × 6.6 mm. Each subarray contains 1,007 circular spatial spots, each with a unique spatial barcode and an approximate diameter of 100 µm; the spots are also arranged with a center-to-center distance of 200 µm. Slide-seq (2019) used DNA-barcoded beads to reduce the spatial resolution to 10 µm. **B.** SPLiT-seq labeled transcriptomes with split-pool barcoding. In each split-pool round, fixed cells or nuclei are randomly distributed into wells, and transcripts are labeled with well-specific barcodes. Barcoded RT primers are used in the first round. Second- and third-round barcodes are appended to cDNA through ligation. A fourth barcode is added to cDNA molecules by PCR during sequencing library preparation. **C.** HDST deposits barcoded poly(d)T oligonucleotides into 2-μm wells with a randomly ordered bead array-based fabrication process and decodes their positions by a sequential hybridization and error-correcting strategy. Three rounds of split-and-pool were performed to produce a bead pool with 65×211×211 different oligonucleotide combinations. **D.** It shows the manner in which the DNA microscopy reaction encodes spatial location. Diffusing and amplifying clouds of UMI-tagged DNA overlap to extents that are determined by the proximity of their centers. UEIs between pairs of UMIs occur at frequencies determined by the degree of diffusion cloud overlap. **E.** DBiT-seq used two sets of barcodes A1-A50 and B1-B50 followed by ligation *in situ* yields a 2D mosaic of tissue pixels, each containing a unique combination of full barcode AiBj (i=1-50, j=1-50).

A recently popular spatial transcriptomic method is capturing the mRNA from tissue sections using a patterned microarray equipped with barcoded oligo-dT primers 7. Such research has demonstrated high-quality RNA sequencing and two-dimensional location information from mouse brain and human breast cancer by placing reverse transcription arrayed primers with distinctive location barcodes on histological sections (**Figure 2A**). In the context of complete tissue sections, positional molecular barcodes in the cDNA synthesis reaction are introduced before the RNA sequence. Fluorescent cDNA is synthesized with Cy3-labeled nucleotides, which are revealed after removing the tissues. This method catches the mRNA in tissue slices with minimal diffusion and then spatial information is revealed by the arrayed oligo-dT primers with location barcodes. Each array device contains a DNA barcode probe including a T7 amplification site, a cleavage site, a spatial barcode, a unique molecular identifier (UMI), oligo-dT VN (V is anything but T and N is any nucleotide), and a sequencing handle. Finally, principal component analysis or t-distributed stochastic neighbor embedding (t-SNE)^15^ and machine learning algorithms for dimensionality decrease and hierarchical clustering are used. Barcoded microplates are used in which the well diameter is 100 µm, enabling each well to capture 10-40 cells. However, the sensitivity of this technique was only 6.9% of single-molecule fluorescence *in situ* hybridization (smFISH). The decoding method of this technology mainly uses sequencing-by-ligation (SBL). Specifically, this technique encodes a unique barcode probe on a microplate and can be decoded before sample preparation to facilitate the interpretation of subsequent results.

Comparing spatial transcriptomics (ST) 7 with the microwell-seq published by Ham in 2018 16, although both use microwell arrays, the former uses a barcoded capture probe and intact tissue for capture, while the latter uses barcode beads to analyze single cells. Each oligonucleotide of the latter consists of a primer sequence, cell barcode, UMI, and a poly-T tail. The barcode beads are synthesized using three split-pool rounds to introduce three parts of oligonucleotides into the microbeads. In the first split cell cycle, carboxyl-coated magnetic beads are randomly distributed into 96-well plates in which 5' amino-modified oligonucleotides are conjugated to the beads. The oligonucleotide in each well has a unique barcode sequence. The beads are then combined and divided into another 96-well plate, where a second barcode sequence is introduced by single-cycle PCR. In the final split-pool round, a third barcode sequence, a UMI, and a poly-T tail are added. After splitting the pool, all oligonucleotides on the same bead will have the same cell barcode but different UMIs, and oligonucleotides on different beads will have different cell barcodes. In general, the encoding method of ST relies on the known probes on microplates and decoding by SBL, while the encoding method of the microwell-seq combines the principle of mixed separation-based technologies.

Stahl made a breakthrough in spatial transcriptomics and presented a protocol that applies spatial transcriptomic technology to mammalian tissues in 2018 6 (**Figure 2A**). The protocol combined histological staining and spatially resolved RNA-seq data from a complete tissue section. Their strategy was based on the ingenious concept of immobilizing cDNA synthesis primers on microscope slides. They assigned primers with a single barcode on a microscope slide to make a microarray. Next, they placed a portion of the tissue on the surface of the microarray where the transcripts in the tissue were reverse transcribed from fixed cDNA synthesis primers. The resulting cDNA library was sequenced and the information was visualized by high-resolution histological imaging of tissue sections. They used the same microplates as before, but each barcoded oligo-dT microarray slide had six sub-arrays 7. The 5' end of each probe is affiliated to a microarray slide and has a segment of deoxy uridine base, which is cleaved after the probe releases. The probe is the key to the encoding of this technology and allows users to locate individual cells back to a spatial position without having to know the expressed genes in advance. Every poly capture segment consists of a UMI, a position barcode, and a library adapter sequence. Significantly, the spatial data is retained by the location barcode. This method can also be combined with the scRNA-seq of cells isolated from adjacent tissue sections 17, 18.

The fundamental mechanism for these NGS-based spatial transcriptomics approaches can be termed barcoded solid-phase RNA capture 19. This is a promising method, and despite the current low resolution and spatial coverage, it enables non-specialized laboratories to perform robust spatial transcriptomics research in tissues. It uses a DNA barcode dot array or barcode bead array to capture mRNA from a fresh tissue section and then lyses to release the mRNA. This method results in a greatly simplified workflow when compared to FISSEQ and seqFISH. The advantage of this technology is that it can analyze the whole mRNA and is easy to apply to most high-quality, fresh-frozen tissues, including clinical sections. Its encoding and decoding methods are relatively simple because the encoded microplates can be decoded *in situ*. This technique is suitable for samples with less structure and a high degree of heterogeneity, such as tumors, while tissues with similar morphological structures between adjacent slices are less suitable. Recently, this method has been applied to clinical diseases. Spatial transcriptomics and single-cell RNA-seq has been combined to investigate pancreatic ductal adenocarcinoma (PDAC). The precise composition of different sub-classifications of tumors varies between individuals and so the sub-population composition and spatial positioning of a given patient may be determined with great prognostic value in the future 20.

### Barcoded bead array-based methods

Slide-seq was developed to reduce the spatial resolution to 10 μm 21 (**Figure 2A**). Slide-seq used 10 μm microparticles named “beads” and *in situ* decoding was performed using sequencing by oligonucleotide ligation and detection (SOLiD). The spatial position of the cells on the slice was determined according to the sequence decoded. Slide-seq can provide a scalable way to obtain spatially resolved gene expression data with near single-cell resolution. Slide-seq uses a self-assembled monolayer membrane of DNA barcode beads on a glass slide to capture mRNA released from a tissue section 21. A “puck” was used to develop high-throughput sequencing for whole genomic expression analysis 22. SOLiD can be used to chemically determine the different barcode sequences of each bead 23. The advantage of Slide-seq is that it can perform spatial analysis of gene expression in frozen tissues and has high scalability and spatial resolution for large tissue volumes. Slide-seq integrates easily with large-scale single-cell sequencing data sets and spatially defines gene expression in both diseased and normal tissues.

However, this emerging method still has several limitations. For example, decoding the DNA barcode bead array is by manual sequential hybridization or SOLiD sequencing, similar to seqFISH, which requires a long and repeated imaging process. In addition, the number of genes detected from Slide-seq data with a resolution of 10-μm is very low and, therefore, it is difficult to visualize the spatial expression of individual genes even when collective gene sets can locate the main types of cells. What's more, these methods are all based on the same mechanism, i.e., barcoded solid-phase RNA capture, and the newly sectioned tissue must be carefully transferred to the network of beads or spots and lysed to release the mRNAs. Although mRNAs are likely to be captured only by the beads or probes, the lateral spread of free mRNAs is inevitable. In addition, the operation of slide-seq is relatively cumbersome, which leads to loss of expression information. The information obtained on a single spot is limited, which will limit the application, and how to extend it to other omics measurements remains unclear.

### Technologies involving mixed separation

In addition to these methods, there is also a scRNA-seq method that uses split-pool to mark the cellular origin of RNA by combining barcode encoding 24. Split-pool ligation-based transcriptome sequencing (SPLiT-seq) does not require dividing individual cells into separate sections, like droplets or microwells, but depends on the cells themselves in sections (**Figure 2B**). The complete workflow before sequencing includes only pipetting steps. SPLiT-seq is compatible with fixed cells or nuclei for efficient sample multiplexing without the need for custom equipment. Each transcriptome is uniquely labeled by encoding a formaldehyde-fixed cell or nucleus suspension through a four-round combination barcode. In every split cell round, fixed cells or nuclei are unsystematically assigned to the wells and the transcripts are marked with well-specific barcodes. Barcode RT primers are used in the first cycle. In the second and third cycles, barcodes are attached to the cDNA by ligation. Through sequencing library preparation, a barcode is added to the cDNA molecule by PCR in the fourth cycle. Lastly, every transcriptome is assembled by combining reads covering the four-barcode combination.

In 2019, high-resolution spatial transcriptomics (HDST) was discovered, which can capture RNA from histological sections on a spatial barcode bead array 25 (**Figure 2C**). Millions of transcript-coupled spatial barcodes are recovered with a resolution of 2-μm, as confirmed in primary breast cancer and mouse brain cancer. Frozen tissue sections are placed on decoded slides, stained, and imaged, and then the RNA is captured and analyzed. To produce high-resolution, high-density bead arrays, a pooling method is used to generate millions of individual barcoded beads, which are unsystematically placed on a hexagonal array of more than 1.4 million 2-μm wells. The position of each bead is decoded by hybridization cycles. Each barcode and bead receives an exclusive spatial color address, which can create an array in a total processing time of ~3 hours. The decoding method proceeds as published before 26, resulting in each well location being encoded with a three-color combination, including FAM, Cy3, and dark. A manufacturing process based on randomly arranged magnetic bead arrays was used to deposit barcoded poly(d)T oligonucleotides into 2-μm wells and their positions were decoded through sequential hybridization and error correction strategies. Simply put, the encoding method of this technology combines the principle of barcoded beads and SPLiT-seq. The main advantage of this technology is the resolution, which is 1400 times higher than ST and 25 times higher than Slide-seq.

There are many kinds of innovative technologies that use the principle of split-pool to encode. For example, prokaryotic expression profile through *in situ* labeled RNA and sequencing (PETRI-seq) 27 uses *in situ* combined indexing to encode barcode transcripts of cells in a single experiment. The technology encodes the barcode through three rounds of 96-well-plate split pools. After the barcode is encoded, the cells are lysed to release the cDNA, which is used for Illumina sequencing. With only a pipetting step and no complicated instruments, a single transcriptome of fixed cells can be uniquely labeled by multiple rounds of splitting, barcode encoding, and merging in microplates. In fact, single-cell combined index RNA sequencing (sci-RNA-seq) 28 and SPLiT-seq 24 are similar in principle. They both rely on cells as part of the barcode, eliminating the need for cell lysis in droplets or microwells, and have the advantages of low cost and high throughput.

Microfluidic deterministic barcoding in tissue for spatial omics sequencing (DBiT-seq) 29 (**Figure 2E**) is a fundamentally new scRNA-seq technology but contains similar decoding steps to mixed separation. The two sets of barcodes A1-A50 and B1-B50 crossed flowing, and are then connected *in situ* to generate a two-dimensional mosaic of tissue pixels. Each mosaic contains a distinct combination of the complete barcode AiBj (i and j are both from 1 to 50). DBiT-seq allows barcode encoding of mRNA, protein, or even other omics on fixed tissue slides at the same time, thus enabling next-generation sequencing to build multi-omics atlas with high spatial resolution. This technology does not require any DNA dot microarrays or decoded DNA barcode bead arrays, but only a series of reagents. It works with existing fixed tissue slides and does not require freshly prepared tissue sections. Barcode A (with fluorophore Cy3) and barcode B (with fluorophore FITC) are combined and the tissue at 50-μm-pixel resolution is imaged through DBiT-seq. The theoretical boundary of the spatial resolution of DBiT-seq is nearly 2-μm and it can simultaneously measure the mRNA transcriptome and fix tissue slides with high spatial resolution in an unbiased manner. Its encoding method uses combined codes, which are divided into vertical and horizontal axes for decoding.

## Physically Segmenting Cells Using Innovative Methods

The most critical step in single-cell research is to effectively capture the single cells of interest. Methods include micro pipetting and microfluidic separation, which often comprehensively depends on mechanical or enzymatic hydrolysis of cell masses or tissues into a single-cell suspension. As a result, any location information of the cell will be lost. Laser capture microdissection (LCM) can accurately capture target cells while retaining the structural and spatial data. Geographical position sequencing (Geo-seq) 30 is the combination of LCM and traditional single-cell RNA-Seq to realize spatial transcriptome sequencing. The spatial transcriptome at single-cell resolution is obtained by the sequencing of a small number of cells with spatial information. Geo-seq has been used to study the spatial transcriptome of early mouse embryos 31, and pathological liver and mouse brains (data not shown) 30. Geo-seq can build a three-dimensional transcriptome atlas using the spatial information to display the transcriptome in space and quantity. Geo-seq can compute the single-cell location address unsystematically using the zip-code mapping protocol. However, this technology only allows transcriptome data to be extracted from a small number of cells while retaining the original spatial information.

There are clear differences between Geo-seq and Slide-seq. The throughput of Geo-seq is relatively low and gene expression information is unknown before LCM. The throughput of Slide-seq is high, and it hybridizes probes with known information to the sample. What's more, Geo-seq uses LCM to capture single cells, which is more sensitive than Slide-seq. GEO-seq is highly operable, but the accuracy of spatial information will be subject to data analysis methods. The sensitivity of Slide-seq is relatively low at only 2.7% (**Table 1**). In order to improve the sensitivity, Slide-seq2 was developed, which increases the sensitivity by an order of magnitude. The enhanced capture efficiency of Slide-seq2 combined with its near single-cell resolution may be used to explore the spatial development of the entire tissue.

## Other Advances in Single-Cell Spatial Transcriptomics

### Progress of assistive tools

Despite the tremendous progress made in the molecular profiling of cellular components relying on optical microscopy or direct physical registration, their spatial localization is still a disconnected and mechanically intensive and specialized process. In 2019, Joshua proposed a DNA microscopy technology that enables scalable non-optical imaging of the relative positions of biomolecules 32. Transcript molecules are labeled *in situ* with random nucleotides by DNA microscopy. Neighboring regions of the molecule from these tandem sequences are decoded by an algorithm. The image of the original transcript is inferred at cellular resolution with precise sequence data. Since its imaging capabilities are derived completely from diffusion molecular dynamics, DNA microscopy establishes a chemically encoded microscope system. The coded microscopy uses a unique event identifier (UEI) (Figure 2D). The extent to which UMI-tagged DNA diffuses and magnifies the cloud depends on the proximity to its center. The UEI between the paired UMIs occurs at a frequency related to the degree of overlap of the diffusion clouds. The frequencies are read by DNA sequencing and inserted into the UEI matrix. Then, UEI is used to infer the UMI location. The DNA microscope can encode the spatial location but it has limited targets and, so far, has not been widely used. There are numerous other advances in this field that are not described here in detail 33. The development of these auxiliary tools will help us to understand and apply single-cell spatial transcriptomics.

### Fluorescence *in situ* hybridization (FISH)-based technologies

The era of spatial transcriptomic dates back to the advances in smFISH 34. By imaging a single RNA molecule in a single cell directly, smFISH can both measure RNA expression quantitatively and provide information of the spatial localization. An individual transcript can be imaged as a diffraction-limited spot using fluorescent microscopy and has been applied to mammal tissues and cells 35, where the cellular subpopulations and spatial heterogeneity can be characterized 36. Cai and colleagues created a smFISH probe library with four unusual versions, each with indistinguishable probe sets 37. These advanced smFISH methods use a multiplexed approach that combines a successive series of hybridization, detection, and pickling to detect thousands of gene expression targets in tissue sections 38. smFISH can detect RNA in its natural spatial setting through construction^39^. Its disadvantage is the high cost and long time. Early attempts were based on multiplex smFISH through spectral barcodes and sequential imaging 40, 41. In the past few years, smFISH has rapidly developed from the detection of a few genes to millions, for example, sequential barcoded fluorescence *in situ* hybridization (seqFISH) 42 and multiplexed error-robust fluorescence *in situ* hybridization (MERFISH) 39, and recently to the entire transcriptome level, for example, SeqFISH+ 42, 43. Compared with the traditional *in situ* hybridization technology, smFISH has higher accuracy and a wider dynamic range and so it can accurately infer spatial information with fewer marker genes. smFISH, and its multiplex variants MERFISH 44 and seqFISH 43, all provide excellent sensitivity and single-cell resolution.

**Figure 3:**
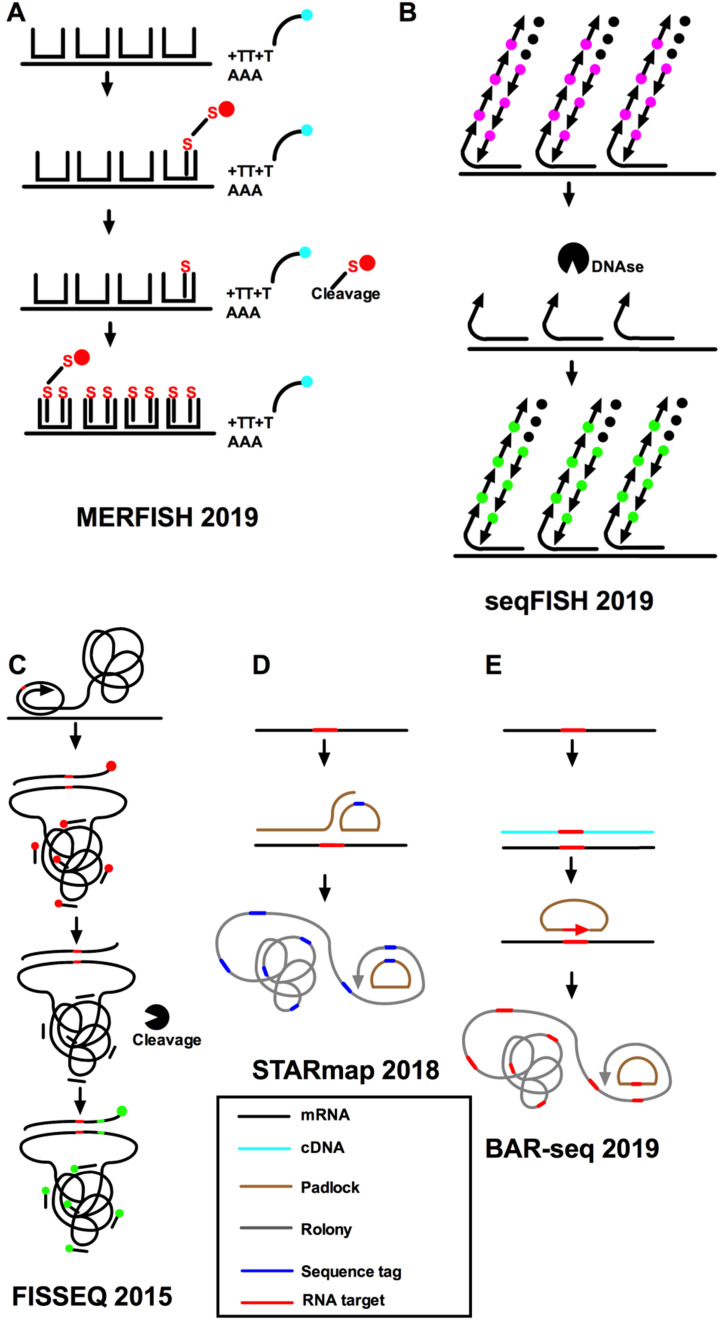
** Imaging-based methods for single-cell spatial transcriptomics. A.** Hybridization of the sample with readout probes complementary to the encoding-probe readout sequences that correspond to the first three bits of the MERFISH barcodes, and imaging of these readout probes in three distinct color channels, cleaving the dye off the readout probe, hybridization and imaging of the next set of readout probes corresponding to the next three bits, and iterate this process until all bits are measured. The RNA signals and registration of decoded RNAs and the immunofluorescence image of the cellular structure are decoded finally. **B.** seqFISH uses temporal barcodes, in which the combination of signal across all cycles is specific to each target. **C.** FISSEQ uses padlock probes to target specific mRNAs, with cDNA synthesis and rolling-circle amplification *in situ*, followed by sequencing by ligation, but reverse-transcribes RNA in an unbiased manner. **D.** STARmap uses sequencing to multiplex read out of hybridization signals. **E.** BAR-seq copies target sequences from the mRNA into the rolonies to allow true sequencing.

The problem of the combination smFISH is the robustness against read errors and MERFISH has been developed to overcome this problem. It ensures a sequence of barcodes for different genes and only multiple read errors could cause assignment errors 39 (**Figure 3A**). Besides, an ingenious scheme of two hybridization stages used by MERFISH lowered the cost of the synthesis of fluorescent probes and the time of hybridization circles. MERFISH assigns error-resistant barcodes to separate RNA species and labels RNA with oligonucleotides representing each barcode. As a result, it can be used for single-cell transcriptome profiling in tissue segments 45. Specifically, MERFISH encodes individual RNA species using error-resistant barcodes and physically imprints barcodes on RNA using combinatorial oligonucleotide labeling, and then measures the barcodes through a series of images of single cells at the transcriptome scale 46. The disadvantages of MERFISH are that it requires special equipment and is relatively expensive. A 69-bit error-corrected coding scheme (23 hybridization rounds and three-color imaging per round) was used 47. The coding scheme includes both the “1” (on) and “0” (off) signal in the barcode and allows the fraction of “1” bits to be changed by altering the number of hybridization circles. When the number of “1” bits of each barcode remains the same, the two strategies (sample expansion or hybridization number increase) will increase the imaging time by achieving the same amount of diluted RNA spot density 38, 42. MERFISH enables the profiling of spatially resolved gene expression of individual cells within a complete biological sample through assigning error-robust barcodes to single RNA species.

A time-barcoded scheme was established to overcome scalability issues that used a series of limited fluorophores. SeqFISH can scale exponentially over time 37, 42 (**Figure 3B**). A color sequence at the known location offers the barcode reading of that mRNA, just as barcode synthesis. The barcodes are identified and quantified by reference to a lookup table to achieve single-cell expression. In addition, single-molecule hybridization chain reaction (smHCR), an amplified version of smFISH, can overcome the auto-fluorescence and scattering in the brain 48, 49. DNase strips the smHCR probe from the target mRNA after imaging, which makes re-hybridization on the same mRNA possible. The color of mRNA can be adjusted through the hybridization probe 50. After hybridization, mRNA is amplified through the addition of complementary hairpin pairs. The round of DNase smHCR is repeated on the similar mRNA to build predefined barcodes, which will change with time. Generally, the hybridization of sequential probes on mRNA in unchanging cells will give an individually predefined color time-series to generate mRNA barcodes *in situ*. At the core of these methods are complex algorithms that can take into account different sources of experimental variability while inferring location information 11,44. This technique encodes the readout probes of different colors and decodes them after a series of hybridization rounds. These methods combine the advantages of two aspects: it can perform a deep unbiased analysis of a large number of individual cells and retain the positional information. The use of combinatorial barcodes can target almost any gene, but its disadvantage is that it requires a large number of probes.

### Barcode *in situ* targeted sequencing methods

To supplement multiplex FISH, *in situ* sequencing (IS-seq) can also be used to perform RNA analysis at the transcriptomic scale of an individual cell. Fluorescence *in situ* sequencing (FISSEQ) can detect millions of RNAs in a non-targeted manner with low efficiency of detection 12, 51 (**Figure 3C**) and may lead to bias due to uneven binding efficiency because of variations in the base structure. Lately, another kind of IS-seq, spatially resolved transcript amplicon readout mapping (STARmap), was developed to significantly increase the efficiency 40 (**Figure 3D**). However, the number of genes that can be detected by fluorescent IS-seq methods is low, and like seqFISH, they require a long time and specialist technology for image processing. The IS-seq method is a complementary technique to reveal intracellular transcriptomes, discover new cell types, identify cells, and to draw maps of cell types within tissues. IS-seq is theoretically like conventional Illumina DNA *in vitro* sequencing. Several researchers have combined microplates or magnetic beads with known barcode sequence information for encoding. Some extremely sensitive multiplex approaches have been put forward, including STARmap 40, IS-seq 52, MERFISH 38, seqFISH 42, and cyclic single-molecule fluorescent *in situ* hybridization 53. All the above approaches lack single nucleotide specificity. In these methods, seqFISH and MERFISH approaches use multiple cycles of hybridization to detect and read multiplex mRNA. STARmap and non-vacuum padlocks use sequencing technologies to multiplex read hybridization signals, or use IS-seq (such as FISSEQ 54) and barista-seq to copy target sequences from mRNA to rolonies for accurate sequencing. Targeted *in situ* RNA sequencing was introduced by using padlock probes to initiate targeted cDNA *in situ* synthesis 52. The padlock method is like the smFISH method because it targets identified genes. All these approaches use nano-balls to amplify the signals, which limit the amount of transcripts and produce a potential bias in favor of certain transcripts. In general, these methods are encoded by means of nanospheres, and all use the improved SBL chemical method to decode the barcode. However, all of the above methods are technically demanding and require advanced image analyzing processes, high sensitivity optical mapping systems, and long imaging workflows for advanced multiplexing 41. What's more, all of them involve a limited set of probes that hybridize to identify RNA sequences and these IS-seq approaches also require custom instruments.

Barcoded anatomy resolved by sequencing (BAR-seq) is an IS-seq method based on an RNA barcode to map the projection of millions of spatially resolved neurons 55. The latest BAR-seq method can be used in conjunction with highly multiplexed FISH or IS-seq methods to predict and correlate the expression of millions of genes. A previously developed sequence-based method was named multiplexed analysis of projections by sequencing (MAP-seq) 56, 57 and enables high-throughput projection mapping. MAP-seq can individually label single neurons with a random RNA sequence or barcode to achieve multiplexing. However, the original MAP-seq protocol depends on tissue homogenization, similar to most other sequencing technologies, and thus it will lose the location information of cells. In this technology, RNAs are converted to cDNAs through reverse transcription, and the cDNAs are then amplified by rolling circle amplification to produce nano-sized balls of DNA called rolonies during the amplification period. The rolonies are sequenced using four fluorescently labeled nucleotides in parallel during the sequencing period. The nucleotide sequence is thus converted into a color sequence and read by a multi-channel fluorescent microscope. BAR-seq combines high-throughput measurements based on cell barcodes, such as high-throughput screening 58, pedigree tracking 59, and projection mapping, with such integrated methods. There are several FISH approaches that read specific genomic tags by sequencing, thus allowing multiplex detection of RNA 38, 48. However, these methods cannot straightforwardly sequence the specific RNA and are not appropriate for the sequencing of barcodes. In a targeted method called Barista-Seq 60 (**Figure 3E**), reverse transcription is used to convert the barcode sequence to cDNA followed by hybridization to a padlock probe, filling in the gaps, and then ligation to form a circular pattern for amplification of the rolling circles. However, this technique has quantity limitations due to prior selection of targets.

## Conclusion and Future Prospects

Recently, transcriptomics has been developed for single-cell RNA analysis and has revolutionized the study of biology and heterogeneity in single cells in cellular immunity, cancer diagnosis, oncology, stem cells, and development 61-63. A number of single-cell RNA sequencing strategies with different strengths and effectiveness have been established 64. However, despite the latest advances in massively parallel scRNA-seq 65, which revealed astonishing cellular heterogeneity in many tissue types 66, 67, the spatial information in the context of tissues is missing from the scRNA-seq data. Therefore, methods have been devised to achieve high-throughput analysis while preserving spatial data about the cellular and subcellular localization.

In this article, we have summarized the latest single-cell spatial transcriptome sequencing technologies and described their encoding methods. There are many types of encoding methods for single-cell spatial transcriptomic sequencing technologies, including microplate-based methods. There are also technologies that use magnetic beads for encoding, split-pool with barcode combination, fluorescent probes for hybridization, padlock probes for *in situ* encoding, and combination encoding methods using unique location signs, like AiBj, for encoding. Although the encoding methods of these technologies are different, their common goal is to bring the resolution close to the single-cell level and to increase throughput. However, we should not pursue greater resolution excessively. Sometimes the resolution is too low to cause undesirable results. Its effectiveness may decrease with the technical resolution and throughput increase. For example, the resolution of HDST is about 2-μm, but its effectiveness is only 1.3%, while the efficiency of ST can be up to 6.9% (**Table 1**). Therefore, we must also consider the issue of efficiency while pursuing increased resolution of novel technologies.

Most technologies, such as ST, slide-seq, Geo-seq, MERFISH, and HDST, can only be applied to fresh-frozen samples and only a few, such as LCM and FISSEQ, can be applied to paraffin samples (FFPE) even though FFPE is the normal way to preserve samples in many laboratories. In a recently published article 68, nanoliter array technology was successfully applied to FFPE. The encoding method of this technology is similar to the combination of ST and FISH.

Heterogeneity of spatial gene expression plays a vital role in biological, pathological, and physiological processes, but genome-wide, high spatial resolution, unbiased biomolecular profiling analysis on large areas of tissue is still a challenge. Our main task in the future will be to develop a simple and cheap method that can simultaneously encode and expand whole genomes with spatial resolution while keeping cells and tissues intact for subsequent target analysis. With this expansion of methods and technology, we are confident that single-cell spatial transcriptomics will rapidly improve our understanding of multicellular tissues and organisms in health and disease.

## Figures and Tables

**Table 1:** Recent technology for single-cell spatial transcriptomics

	Published time	Target	Single cell	Spatial resolution	Coding method	The type of technology	Efficient	Cost
FISSEQ	2015	RNA	Yes	subcellular	Padlock probe	Barcode *In situ* Targeted	0.005%	NA
Geo-seq	2017	RNA; DNA	Yes	cellular	zip-code mapping	Microdissected based	NA	NA
SPLiT-seq	2018	RNA	Yes	cellular	Four barcode combination	Mixed separation based	NA	$0.01/cell
STARmap	2018	RNA	Yes	subcellular	Barcoded SNAIL probes	Barcode *In situ* Targeted	5-40%	NA
seqFISH	2018	RNA	Yes	subcellular	Probe hybridization	*In situ* Hybridization based	84%	expensive
merFISH	2019	RNA	Yes	subcellular	Probe hybridization	*In situ* Hybridization based	80%	expensive
ST	2016; 2018	RNA	Yes	cellular, 100 µm	Barcoded microarray slide	Microwell plate based	6.9%	~$650/plate
Slide-seq	2019	RNA	Yes	cellular, 10 µm	barcoded beads	Barcoded beads based	2.7%	~$200 to $500 for the pucks
HDST	2019	RNA	Yes	subcellular, 2 µm	barcoded beads	Barcoded beads and mixed separation based	1.3%	NA
DBiT-seq	2019	RNA; DNA, protein	Yes	cellular, 10 µm	AiBj	Mixed separation based	NA	NA
BAR-seq	2019	RNA; DNA, protein	Yes	cellular	rolony	Barcode *In situ* Targeted	30%	$50 neurons per cortical area
PETRI-seq	2020	RNA	Yes	cellular	*In situ* combinational indexing	Mixed separation based	2.5~10%	$0.056/cell

## Abbreviations

scRNA-seq: single-cell RNA sequencing
UMI: unique molecular identifier
t-SNE: t-distributed stochastic neighbors embedding
SBL: sequencing-by-ligation
PDAC: pancreatic ductal adenocarcinoma
SOLiD: sequencing by oligonucleotide ligation and detection
NMFreg: Non-Negative Matrix Factorization Regression
SPLiT-seq: Split-pool ligation-based transcriptome sequencing
HDST: high-resolution spatial transcriptomics
PETRI-seq: prokaryotic expression profile through *in situ* labeled RNA and sequencing
sci-RNA-seq: single-cell combined index RNA sequencing
DBiT-seq: Deterministic Barcoding in Tissue for spatial omics sequencing
LCM: Laser capture microdissection
Geo-seq: geographical position sequencing
UEI: unique event identifier
FISH: fluorescence *in situ* hybridization
smFISH: single-molecule fluorescence *in situ* hybridization
seqFISH: sequential barcoded fluorescence *in situ* hybridization
MERFISH: Multiplexed error-robust fluorescence *in situ* hybridization
smHCR: single molecule hybridization chain reaction
IS-seq: *in situ* sequencing
FISSEQ: Fluorescence *in situ* sequencing
STARmap: spatially resolved transcript amplicon readout mapping
BAR-seq: Barcoded anatomy resolved by sequencing
MAP-seq: multiplexed analysis of projections by sequencing
FFPE: paraffin-embedded
ST: spatial transcriptomic
